# Orientation in multi-layer chitosan hydrogel: morphology, mechanism, and design principle

**DOI:** 10.1038/srep07635

**Published:** 2015-01-06

**Authors:** Jingyi Nie, Wentao Lu, Jianjun Ma, Ling Yang, Zhengke Wang, An Qin, Qiaoling Hu

**Affiliations:** 1 MOE Key Laboratory of Macromolecular Synthesis and Functionalization, Department of Polymer Science and Engineering, Zhejiang University, Hangzhou, China; 2 Department of Orthopaedics, Sir Run Run Shaw Hospital, School of Medicine, Zhejiang University, Hangzhou, China; 3 Department of Orthopedics, Shanghai Key Laboratory of Orthopedic Implants, Shanghai Ninth People's Hospital, Shanghai Jiaotong University School of Medicine, Shanghai, China

## Abstract

Hydrogels with organized structure have attracted remarkable attentions for bio-related applications. Among the preparation of hierarchical hydrogel materials, fabrication of hydrogel with multi-layers is an important branch. Although the generation mechanism of layers had been fully discussed, sub-layer structure was not sufficiently studied. In this research, multi-layered chitosan hydrogel with oriented structure was constructed, and the formation mechanism of orientation was proposed, based on gelation behavior and entanglement of polymer chains in the hydrogel-solution system. Employing the layered-oriented characteristic, chitosan hydrogel materials with various shapes and structure can be designed and fabricated.

Hydrogels are three-dimensional macromolecular networks cross-linked by chemical or physical interactions, with their applications pervading daily life and advanced industry^1^. Hydrogels, in most cases, are homogeneous materials and are considered for their bulk properties^2^. However, hierarchical soft material exhibiting organization at different scales can be a prerequisite for bio-related applications^3^,^4^,^5^,^6^,^7^. The control of hydrogel structure, therefore, has been extensively studied to meet various needs in specialized fields. Thus the understanding of gelation mechanisms possesses fundamental importance for the design of organized hydrogel with enhanced mechanical and functional performance^8^,^9^.

Among materials utilized in preparation of hydrogels, chitosan (CS), a polysaccharide obtained from the deacetylation of chitin, has received considerable attention for its intrinsic properties: biocompatibility, biodegradability, bacteriostatic effects and abundance in nature^10^,^11^,^12^. The modulation of CS hydrogel structure relies on the exploration of formation mechanisms. Although the study of structure and interactions in CS hydrogels sheds light on formation mechanisms on molecular level, the construction of highly sophisticated microstructure remains challenging^13^,^14^,^15^. Several methods to fabricate hydrogels with multi-layer structure have been reported, focusing on generation mechanisms of separated spatial patterns^16^,^17^,^18^,^19^. Besides layered architectures, structure within each layer has been observed^17^, yet its further study is deficient. The understanding of sub-layer structure is essential for the application of CS hydrogel, since it contributes to interesting properties including mechanical strength and morphology of internal surface^20^,^21^.

In this research, oriented structure was constructed in multi-layered CS hydrogel without any auxiliary crosslinking agent. Gelation process of solubilized CS in acidic aqueous medium was employed. CS becomes a polyelectrolyte because of the protonation of –NH_2_ groups^22^. And the CS solution can be transformed into hydrogel when it comes in contact with alkali. Generally, the soluble-insoluble transition of CS occurs around pH 6–6.5, and hydrogel will be formed at higher pH^23^. The formation of layers can be explained by the Liesegang ring phenomenon, which is caused by periodical precipitation in the supporting medium by the encounter of an inner and an outer electrolyte^24^,^25^,^26^. And in this system, CS played dual roles of the inner electrolyte and the supporting medium. The introduction of orientation endowed multi-layered hydrogel with order in another direction. The present work focused on structural characteristics in 3-dimensions and the formation mechanism of orientation. Then the mechanism could be generalized to design CS hydrogel materials with various shapes and structure.

## Results

### Gelation process and interface

**Figure 1:**
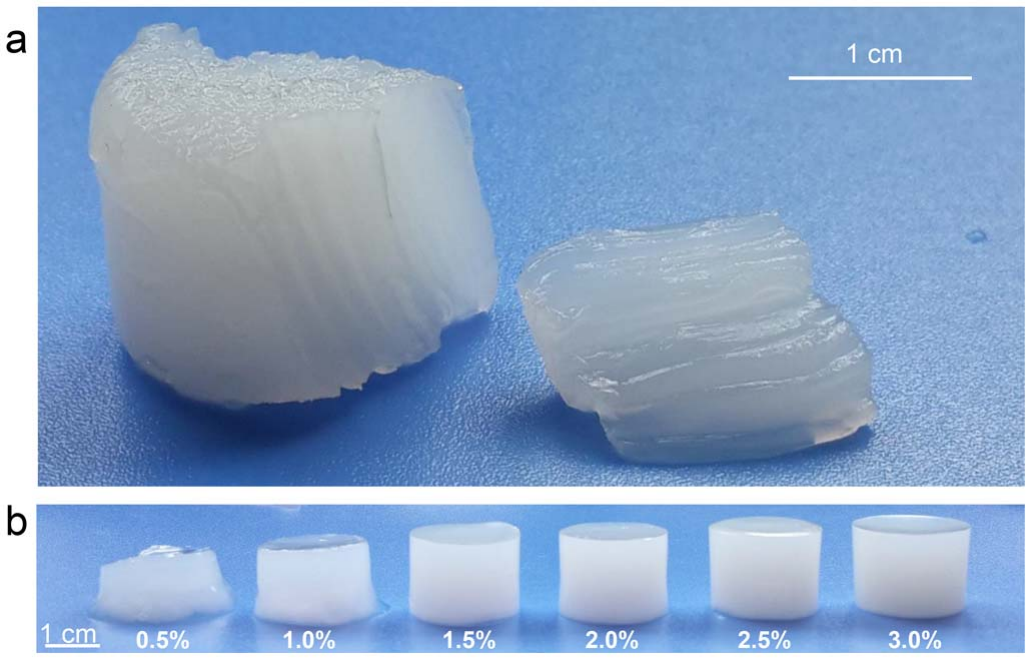
Digital photographs of hydrogel prepared by single opening mold. (a) Longitudinal section of hydrogel, *c*(CS) = 3.0 wt.%; (b) CS hydrogels with different *c*(CS).

Figure 1 presented CS hydrogel prepared by single opening mold. Orientation can be observed from the longitudinal section on both macro-and microscopic level (Fig. 1a, Supplementary Fig. S1 and S2). The direction of orientation was not arbitrary, but possessed a regular pattern.

**Figure 2:**
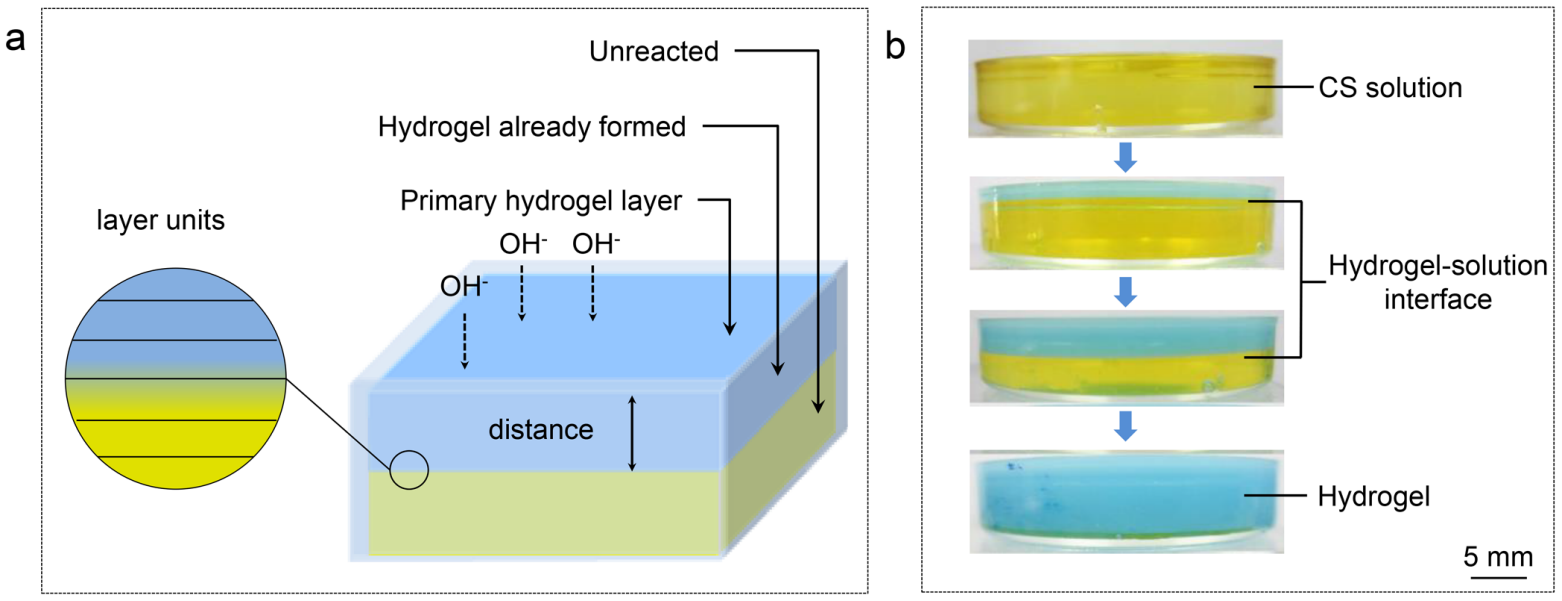
Preparation of chitosan hydrogel by a single opening mold. (a) Schematic diagram of preparation; (b) Digital photographs of gelation process at different reaction time. Solution and hydrogel were colored by bromothmol blue for visibility. In view of pH range, the blue part corresponded to hydrogel already formed while the yellow part corresponded to unreacted solution, and the boundary indicated the hydrogel-solution interface.

Gelation process of CS solution was recorded by digital photographs (Fig. 2b). The single opening mold was selected to simplify the analysis of diffusion. Gelation started as soon as the surface of CS solution contacted with coagulation bath. The contact area turned to a hydrogel layer immediately (denoted as the primary hydrogel layer), and served as the interface between the system and OH^−^ source. Hydrogel-solution interface advanced from the primary hydrogel layer to the bottom of the mold. Instead of forming cross-links in the whole system simultaneously^11^,^13^,^14^, OH^−^ diffused into the system to generate gelation gradually. So gelation process of this system possessed a layer-wise characteristic. It had been proved that this characteristic enabled the formation of multi-layers^4^. Besides, this characteristic endowed the system with directivity, which laid the foundation of orientation.

### Structural characteristics of hydrogel

**Figure 3:**
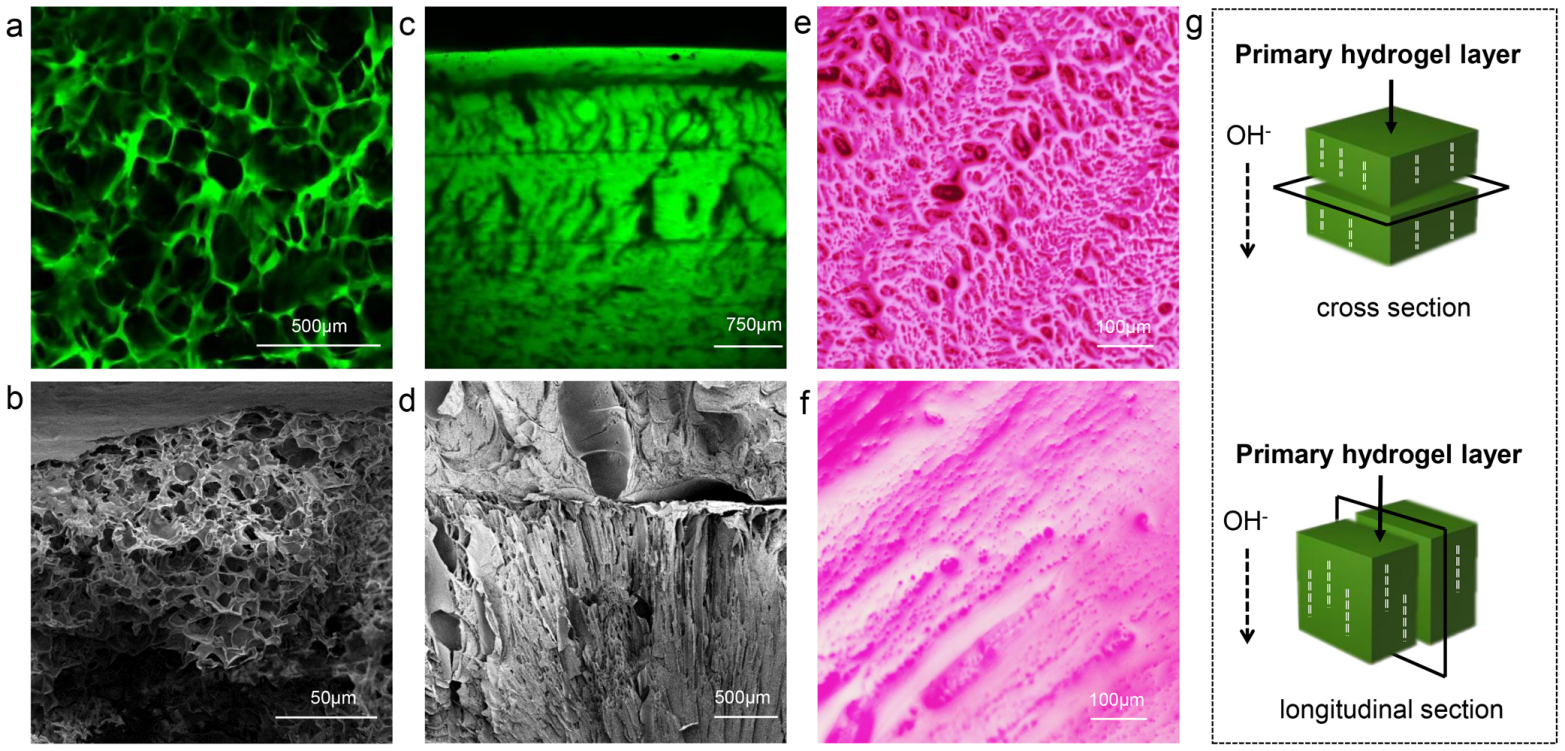
Morphology of hydrogel prepared by single-opening mold. (a)–(f) Micrographs of hydrogels: (a) CLSM image, *c*(CS) = 0.1 wt.%, cross section; (b) SEM image, *c*(CS) = 0.5 wt.%, longitudinal section; (c) CLSM images, *c*(CS) = 4.0 wt.%, longitudinal section; (d) SEM images, *c*(CS) = 3.0 wt.%, longitudinal section; (e) and (f) Fluorescence microscope images, bright field, colored by rhodamine for visibility, cross section and longitudinal section, respectively, *c*(CS) = 3.0 wt.%; (g) Schematic diagram of longitudinal and cross section of hydrogel, the longitudinal section is perpendicular to the primary hydrogel layer, while the cross section is parallel to it.

**Figure 4:**
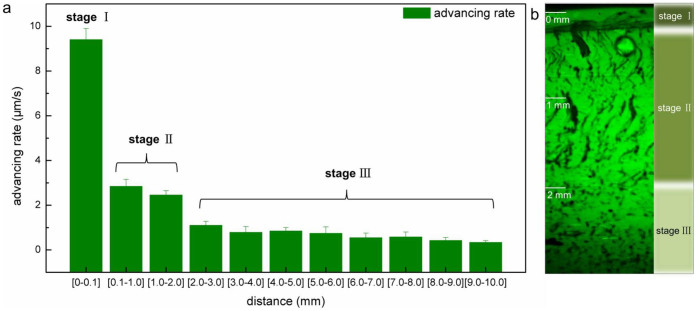
Three stages of gelation process. (a) Average advancing rate of interface at different distance regions, error bars indicate standard errors for n = 5, (b) CLSM image of corresponding hydrogel on the longitudinal section.

**Figure 5:**
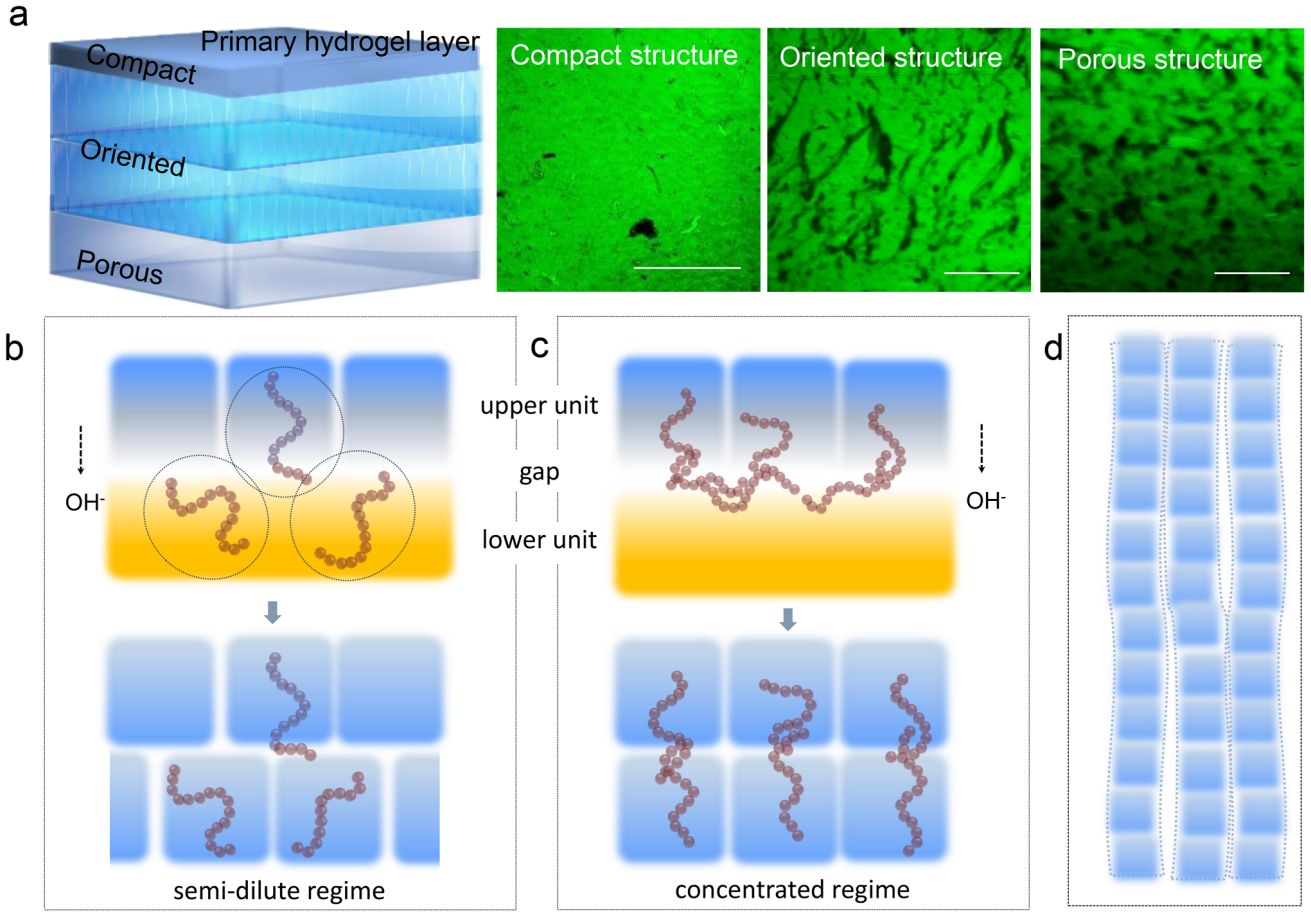
Schematic model of layered-oriented CS hydrogel and illustration of formation mechanism. (a) Schematic model, CLSM images corresponding to compact region, oriented region and porous region, respectively, *c*(CS) > 1.0 wt.%, scale bar represented 100 μm. (b)–(d) Macromolecular interactions in “gel-sol consecutive reaction units” during the gelation process, (b) semi-dilute regime, (c) concentrated regime, and (d) formation of oriented structure by stacking of reaction units.

CS hydrogels with different *c*(CS) were prepared by single opening mold, and the morphologies of hydrogels were shown in Fig. 3. In hydrogels with different *c*(CS), orientation cannot always be observed. To be more specific, these hydrogels presented two types of structures. The first type corresponded to hydrogels with *c*(CS) < 0.5 wt.%. In these samples, an ordinary random 3D network was formed (Fig. 3a and b), which could be considered isotropic. However, when *c*(CS) was higher than 1.0 wt.%, a different type could be observed (Fig. 3 c, d, e and f). The second type possessed complexity in three dimensions, which was summarized as following: 1) Multi-layered structure. Spatially separated layers had been observed (Fig. 3c and d), which was consistent with Liesegang ring phenomena. The number and thickness of layers showed no deviation from previous studies, and would not be further discussed in this research^17^,^18^,^19^; 2) Orientation. Oriented structure had been observed in the hydrogel (Fig. 3c, d, and f). Interestingly, orientation appeared along the direction of OH^−^ diffusion and joined every layer at about 90°. This phenomenon confirmed the relationship between orientation and the direction of diffusion. 3) Structural transition with increased distance to the primary hydrogel layer (Fig. 3d). The region close to the primary hydrogel layer presented a smooth and compact structure. At longer distance, oriented region appeared and then transformed into a loose region, which had porous structure. Transition between compact and oriented region was demonstrated in Supplementary Fig. S3, and transition region between the oriented and the porous structure could be observed in Fig. 3d and Fig. 4b. According to the characteristics mentioned above, this typical structure could be referred to as a layered-oriented structure, which was schematically illustrated in Fig. 5a.

### Formation mechanism of orientation

**Figure 6:**
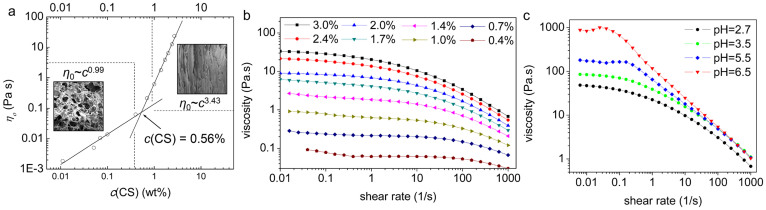
Rheological properties of CS solution. (a) Dependence of zero-shear viscosity (*η_o_*) on *c*(CS) at 25°C. Insets are SEM images corresponding to *c*(CS) < 0.5 wt.% and *c*(CS) > 1.0 wt.%, respectively; (b) Dependence of the steady shear viscosity on the shear rate for CS solutions with various concentrations at 25°C, the critical shear rates are as follows: 0.2 ~ 0.4 s^−1^, 1.0 ~ 2.5 s^−1^, 4 ~ 6 s^−1^, 7 ~ 10 s^−1^, 25 ~ 39 s^−1^, 63 ~ 100 s^−1^, 130 ~ 158 s^−1^, which were determined by first order derivative; (c) Dependence of the steady shear viscosity on the shear rate for CS solution at different pH, including unreacted CS solution (pH = 2.7) and CS solution near gelation pH (pH = 6.5).

In order to control and design the structure of hydrogels, a better understanding of the layered-oriented structure is needed. So the formation mechanism of hydrogel structure was explored. Orientation only appeared in hydrogels with relatively high *c*(CS). This indicated that polymer concentration played an important role in the formation of hydrogel. Essentially, polymer concentration is closely related to interactions between macromolecular chains in the solution. To understand the interactions between macromolecular chains in the solution, rheological properties of CS solution were studied and the results were shown in Fig. 6.

Based on the layer-wise characteristic, the system could be considered as numerous layer units stacked along the diffusion direction of OH^−^ (Fig. 2a). Every layer was thin enough so gelation inside one unit was approximately simultaneous. Units above the gel-sol interface were inactive since they had already finished gelation, while units below that had not been activated. As a result, two layer units just across the gel-sol interface were taken into consideration: gelation reaction had just finished in the upper unit and was about to start in the lower unit. For macromolecules in the upper unit, part of their chains were deprotonated and embedded in the hydrogel, while rest of the chains were still in the solution and interacted with macromolecules in the lower unit. Their interaction could be analyzed in the view of entangle-disentangle equilibrium. The critical shear rate (γ_c_) corresponding to transition from Newtonian to shear-thinning behavior were shown in Fig. 6b. The emergence of shear thinning in solutions is most likely due to the fact that recently disentangled polymers have insufficient time to re-entangle with new neighbours^31^,^33^. So the critical shear rate reflected entangle-disentangle equilibrium in the solution, which shifted to a higher shear region with an decrease of *c*(CS). This indicated that in less concentrated solutions, destruction of original state of entanglement happened more quickly (Supplementary Fig. S4).

The dependence of zero-shear viscosity (*η_o_*) on *c*(CS) was demonstrated in analogous plot (Fig. 6a). Generally, polymer solution can be divided into three concentration regimes, *i.e.*, dilute, semi-dilute and concentrated regimes. The boundary between dilute and semi-dilute regimes is the overlap concentration (c*), while that for semi-dilute and concentrated regimes is the entanglement concentration (*c_e_*). Transition between two regimes is usually accompanied by a remarkable change in the *η_o_* dependence of concentration^27^,^28^. In this case, the plot fell in two linear regions, with an abrupt change of slope from 0.99 to 3.43. The two linear regions had an intersection, and the corresponding concentration (0.56 wt.%) was denoted as the concentration of intersection (*c*_i_). According to aforementioned theories, *c_i_* could be either c* or *c_e_* of CS solution. The possibility of *c_i_* being the overlap concentration (c*) could be ruled out. Because empirically, for polymer with M ≈ 10^5^ Da, the c* is about 10^−4^ wt.%. The higher the molecular weight is, the lower the c* will be^29^. For CS used in this research (M*_η_* = 2.1 × 10^6^), the c* would correspond to *c*(CS) lower than 10^−4^ wt.%. So it could be concluded that *c_i_* was the *c_e_* of CS solution. This could be further confirmed by previous study on c* and *c_e_* of chitosan solutions^30^.

Thus, a formation mechanism of oriented structure was proposed based on macromolecular interaction and the diffusion direction of OH^−^. When contacting with OH^−^, originally homogeneous CS solution turned to hydrogel containing CS-rich micro-zones and water-rich micro-zones based on phase equilibrium of polymer solution^31^. For CS solution in concentrated regime, in which fully interpenetrated macromolecular networks had been established, the motion of polymer chains in the lower unit was restricted due to entanglement in the gap. In the gel-sol consecutive units, due to concentration gradient of OH^−^, the gap had higher pH. When pH of CS solution reached gelation point, the viscosity of system was higher and transition from Newtonian to shear-thinning behavior happened at lower frequency (Fig. 6c). Thus, polymer chains entangled in the gap had longer relaxation time than those in the lower unit (Supplementary Fig. S5). So macromolecules in lower unit relaxed from original entanglement with restriction maintained in the gap. As a result, macromolecules were influenced by the previous unit and rearranged below CS-rich micro-zones rather than water-rich micro-zones. With proper gelation time, the organized rearrangement can be “frozen” in the lower unit (Fig. 5b). But for CS solution in the semi-dilute regime, in which polymer chains were not sufficiently entangled, restriction to the next unit could be quickly eliminated. So the arrangement of CS-rich micro-zones in the lower unit was independent from that in the previous unit (Fig. 5c). This whole gelation process could be considered as stacking of numerous units, which enabled by layer-wise gelation process. When the organized stacking of CS-rich micro-zones along the OH^−^ diffusion direction was achieved, orientation would appear in the hydrogel consequently (Fig. 5d). This was in good agreement with the transition concentration between random network and orientation structure. The random network structure appeared in the semi-dilute regime, while the layered-oriented structure was formed in the concentrated regime.

### Structural transition

The formation of orientation required proper gelation time to “freeze” organized rearrangement in the lower unit. However, diffusion of OH^−^ from the coagulation bath to the interface decreased by the increasing thickness of hydrogel^32^. So the gel-sol interface did not advance from the primary hydrogel layer to the bottom of mold at a constant speed (Fig. 4), indicating that gelation rate of every unit decreased.

The structural transition along the diffusion direction showed accordance to the change of advancing rate. The highest advancing rate corresponded to 0–0.1 mm region and the compact structure. The 0.1–2 mm region corresponded to oriented structure, showing a decrease in advancing rate. The decrease of advancing rate at the beginning of 2–3 mm region corresponded to the transition to the porous structure.

The transition from one structure region to another is in accordance with this mechanism. The whole gelation process of this system could be divided into three stages based on the relationship between gelation time and the time to achieve organized rearrangement (Supplementary Fig. S6). In stage I, due to high gelation rate, macromolecules in the lower unit were “frozen” even before organized rearrangement was achieved. In stage II, with decreased gelation rate, requirements for organized rearrangement and stacking were satisfied. When gelation rate further decreased in stage III, original entanglement in the gap relaxed and eliminated restriction of macromolecular motion in the lower unit. This indicated the proper diffusion rate of OH^−^ is essential for orientation. Longer oriented region could be achieved with higher *c*(NaOH) in coagulation bath which was in good agreement with the mechanism (Supplementary Fig. S2).

**Figure 7:**
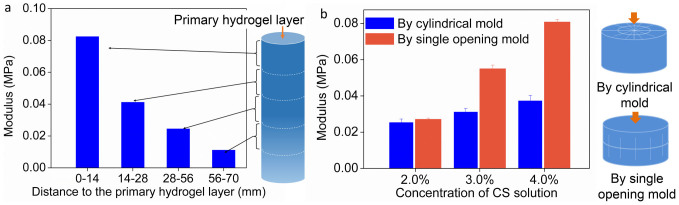
Mechanical property of CS hydrogels. (a) Modulus of hydrogel in different distance range, *c*(CS) = 4.0 wt.%; (b) Modulus of hydrogel samples prepared by different molds, which possessed different directions of orientation, *c*(CS) = 3.0 wt.%; Schematic showing the orientation in hydrogel samples, and relative positions of orientation to the direction of compression. Error bars indicate standard errors for n = 3.

From another perspective, entanglements of macromolecular chains between two consecutive units become weaker with longer gelation time. So hydrogel formed in stage I showed the most compact structure, while porous structure formed in stage III was the loosest structure in all three regions. Transition of structure was also supported by mechanical property. Compression tests indicated that, the modulus of hydrogel decreased with the increase of distance to the primary hydrogel layer (Fig. 7a). The relationship between entanglement and modulus was also supported by the mechanical tests on hydrogels with different *c*(CS) (Fig. 1b and Supplementary Fig. S7).

### Design of hydrogel structure

**Figure 8:**
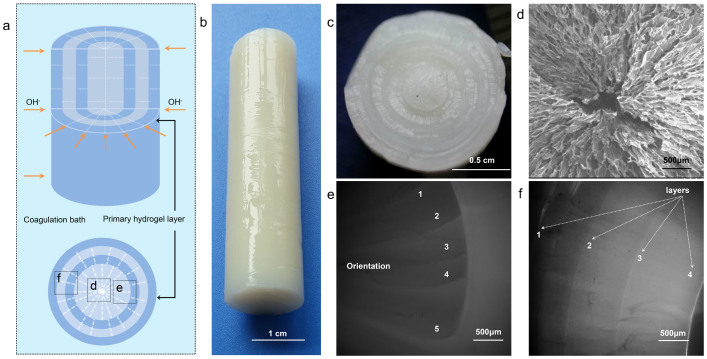
Hydrogel prepared by cylindrical mold. (a) Schematic diagram of preparation of hydrogel by cylindrical mold; (b)–(c) Digital photographs of hydrogel, overall view and cross section, respectively; (d)–(f) Morphology of hydrogel at different positions; positions were marked out in (a) with corresponding letters: (d) Center of radial pattern, SEM images of hydrogel after freeze-drying; (e) Orientation in radial pattern, CLSM image, bright field; (f) Concentric layers, CLSM image, bright field.

Hydrogel prepared by single opening mold was an illustration of structural characteristics of this system. Hydrogels with more complicated architecture could be fabricated based on this mechanism by cylindrical mold (Fig. 8 a and b). In these hydrogels, the layered-oriented structure could be clearly seen (Fig. 8 c, d, e, and f). Layers were in the form of concentric circles, which were all parallel to the primary hydrogel layer. And orientation presented a radial pattern, which was along the diffusion direction of OH^−^. The morphology of hydrogel prepared by cylindrical mold confirmed the structural characteristics discussed above.

The direction of orientation is very important in the design and application of hydrogel. The direction of orientation exerted influence on mechanical property in a certain direction, which was demonstrated in Fig. 7b. The direction of compression was parallel to the orientation of samples prepared by single opening mold, while it was perpendicular to the orientation of samples prepared by cylindrical mold (Fig. 7c). The former showed higher modulus with same *c*(CS). With increase of *c*(CS), orientation became stronger and the influence of enhancement became more apparent. However, design of the latter was still meaningful, since it met the requirements of other applications. Because multi-layers stopped crack propagation to improve the bending strength^35^, and orientation along the radial direction improved the compressive strength^36^,^39^,^40^. Chitosan rod prepared via this hydrogel had been studied as an internal fracture fixation material, which showed good bending strength^34^,^35^,^36^,^37^,^38^.

## Discussion

The gelation process of solubilized CS in acidic aqueous medium possesses a layer-wise characteristic, which contributes to unique layered-oriented structure in resulted hydrogel. Hydrogels prepared this way share structural characteristics: multi-layer structure parallel to the isopleths of *c*(OH^−^), and oriented structure formed along the diffusion direction of OH^−^. Sufficient entanglement of macromolecular chains and proper diffusion rate of hydroxyl ions are two vital requirements in the formation of organized structure.

Based on the structural characteristics, various hydrogels with different shapes could be prepared (Supplementary Fig. S8). And hydrogel prepared by this system showed good biocompatibility (Supplementary Fig. S9, S10, and S11). Besides the utilization of multi-layer structure, there are two principles to facilitate the design of hydrogel. 1) Orientation along specific direction could be used to enhance mechanical performance, or modulate the transportation of substance. 2) The material possesses structural transition and consequent modulus gradient, which could serve as a modulate signal in applications like cell migration^41^,^42^,^43^.

Moreover, CS cannot be manufactured by screw extruder due to the multiple hydrogen-bonds between macromolecular chains, so CS-based materials are generally produced from CS solution. Hydrogel prepared by this system offered an effective way to fabricate CS-based materials with sophisticated structure.

## Methods

### Materials

CS was purchased from Zhejiang Gold Shell Pharmaceutical Co. Ltd. The average viscosity molecular weight (M*_η_*) of CS is 2.1 × 10^6^ Da, and degree of deacetylation (DD) is 75.4%. Fluorescein iso-thiocyanate (FITC) was purchased from Sigma Chemical Company. Fluorescein iso-thiocyanate-labeled CS (FITC-CS) was prepared following the process reported in literature^44^. Sodium hydroxide and acetic acid were purchased from Sinopharm Chemical Reagent Co., Ltd.

### Preparation of CS hydrogel

Coagulation bath was 10 wt.% NaOH aqueous solution. The volume of NaOH (aq) was much larger than that of CS solution, so the *c*(OH^−^) could be considered as constant. CS solution with desired concentration, *i.e. c*(CS), was prepared by dissolving CS powder in 2 vol.% acetic acid aqueous solution. Two types of hydrogels were prepared, by single opening mold and semipermeable membrane cylindrical mold, respectively. CS solution was filled in the mold and immersed in the bath till the gelation was completed. (Fig. 2a and Fig. 8a). CS hydrogels were washed with deionized water repeatedly to be neutral. For the interest of simplicity, “hydrogel prepared from CS solution with *c*(CS) = x wt.%” was shorten as “hydrogel with *c*(CS) = x wt.%”.

### Morphology observation of CS hydrogel

CS hydrogels were prepared with different *c*(CS) (0.1 wt.%–4.0 wt.%). These samples were studied by scanning electron microscopy (SEM), confocal laser scanning fluorescence microscope (CLSM), and fluorescence microscope. For SEM observation, hydrogels were freeze-dried and then gold-sprayed for conductance. While for confocal fluorescence microscopy characterization, hydrogels were prepared with FITC-CS and kept wet for observation. HITACHI S-4800 SEM, Leica TCS SP5 CLSM, and OLYMPUS DP72 were used for the observation.

### Rheological properties

Concentration of the CS solutions ranging from 0.01–3.00 wt.% were prepared and characterized for their rheological properties. Rheological measurements were performed on AR-G2 rheometer (TA Co., USA). The pH of gelation point of solution *c*(CS) = 3.0 wt.% was determined by titration, monitored by a PHS-3C precise pH instrument (Shanghai scientific instrument co., ltd.)

### Hydrogel formation rate

Before the gelation completed, there existed an interface between hydrogel already formed and unreacted CS solution. The interface kept advancing along with the gelation process. Advancing rate was defined as the distance of the interface advanced per second. For better observation of the interface, an indicator, bromothmol blue, was added into CS solution. Advancing rate was calculated by reaction time and position of the interface. The experiments were repeated five times and *c*(CS) is 3.0 wt.%.

### Mechanical tests

The mechanical property of CS hydrogels were obtained on a universal materials testing machine (Instron, 5543A) at a strain rate of 2% min^−1^ for compression tests at room temperature. Hydrogels were prepared to be cylinder samples.

## Author Contributions

J.Y.N., Z.K.W. and Q.L.H. conceived and designed the research, analysed the experimental data and wrote the paper; J.Y.N., W.T.L. and L.Y. conducted the experiments; Z.K.W. and Q.L.H. supervised and directed the project. J.J.M. and A.Q. completed the cell experiments.

## Supplementary Material

Supplementary Information Supplementary information
