# Supplementary material for: Orientation in multi-layer chitosan hydrogel: morphology, mechanism, and design principle
Source: Sci Rep. 2015 Jan 6;5:7635. doi: 10.1038/srep07635 (PMC4284508; doi:10.1038/srep07635)
Supplement: Supplementary Information [file srep07635-s1.pdf]

Supplementary information

Orientation in multi-layer chitosan hydrogel: morphology, mechanism, and design principle

Jingyi Nie[1], Wentao Lu[1], Jianjun Ma[2], Ling Yang[1], Zhengke Wang[1]*, An Qin[3]* & Qiaoling Hu[1]*

[1] MOE Key Laboratory of Macromolecular Synthesis and Functionalization, Department of Polymer Science and Engineering, Zhejiang University, Hangzhou 310027, China.

[2] Department of Orthopaedics, Sir Run Run Shaw Hospital, School of Medicine, Zhejiang University, Hangzhou 310016, China.

[3] Department of Orthopedics, Shanghai Key Laboratory of Orthopedic Implants, Shanghai Ninth People's Hospital, Shanghai Jiaotong University School of Medicine, Shanghai 200011, China.

Correspondence and requests for materials should be addressed to Z.K.W.(email: wangzk@zju.edu.cn); A.Q.(email: dr.qinan@gmail.com); Q.L.H.(email: huql@zju.edu.cn).

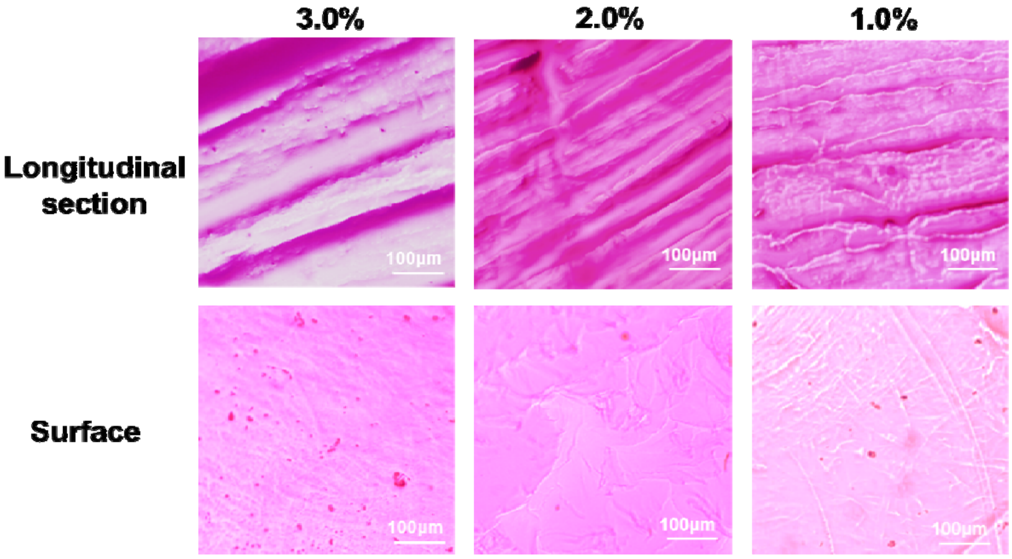

Supplementary Figure S1. Longitudinal section and surface structure of CS hydrogel with different *c*(CS), fluorescence microscope images, bright field, colored by rhodamine for visibility.

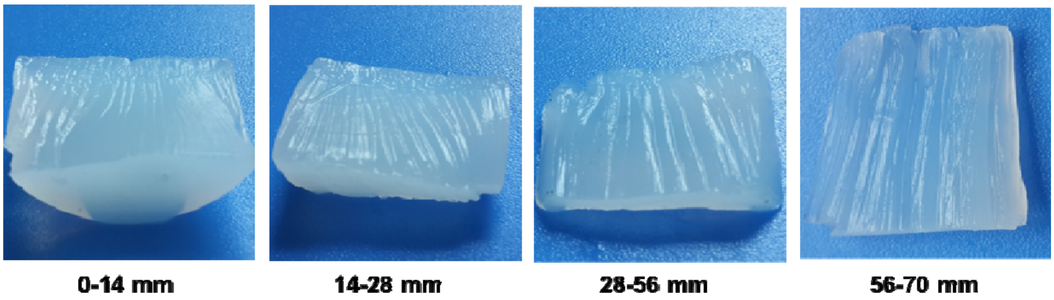

Supplementary Figure S2. Longitudinal section of hydrogel at different distance to the primary hydrogel layer, *c*(CS)=4.0 wt.%;

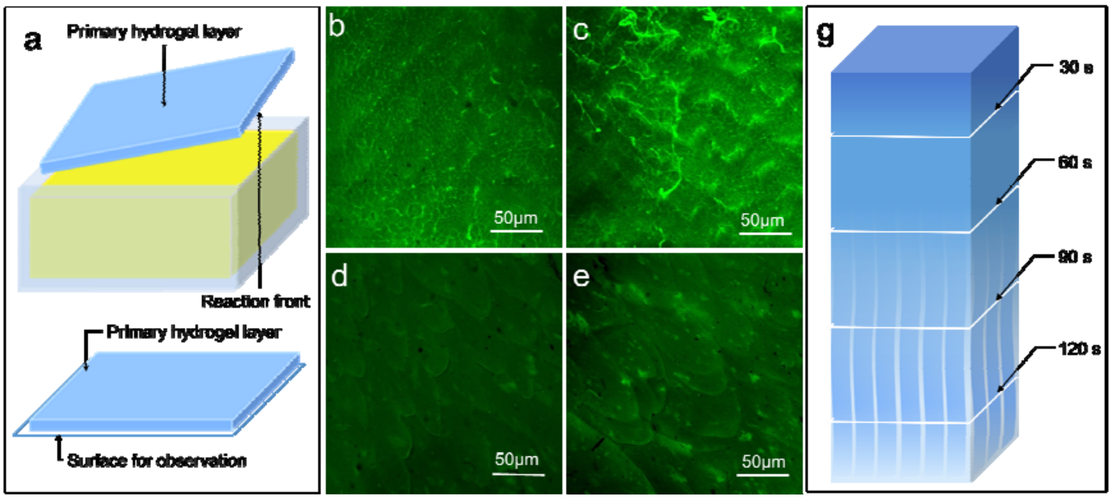

Supplementary Figure S3. Transition between compact region and oriented region. (a) Schematic diagram of sample preparation for the observation of interface. (b-e) CLSM images of hydrogel morphology at the gel-sol interface, terminated at a certain time after the gelation process began: (b) 30 s, (c) 60 s, (d) 90 s, (e) 120 s; (g) Schematic of the relationship between time evolution of the structure and structural transition in the hydrogel.

To observe the structural transition between compact region and oriented region, confocal fluorescence microscopy was utilized to study hydrogel samples, which were prepared with FITC-CS. The *c*(FITC-CS) of the polymer solution was 3 wt.% in this section. The preparation of hydrogel samples was described below. Firstly, preparation of a plate-shaped sample was started. After a short period of time, the gelation process was terminated by separating hydrogel already formed from unreacted FITC-CS solution. The separated hydrogel was immediately washed to remove the residual FITC-CS solution. This step was performed meticulously without touching the surfaces of separated hydrogel. The thickness of hydrogel is related to the gelation time. When the gelation process was terminated at different gelation time (30s, 60, 90s, 120s), the observed surface reflected the cross section of hydrogel with different thickness, *i.e.* different distance to the primary hydrogel layer. Experiment in this section was equivalent to preparing slices of hydrogel on the cross section (*Supplementary Fig. S3g*). It can be observed from Supplementary Fig. S3b that, the cross section of hydrogel was smooth. However, with the increase of thickness, the cross section of hydrogel became rough and showed horizontal cut of fibrous structure. This indicated the appearance of oriented region.

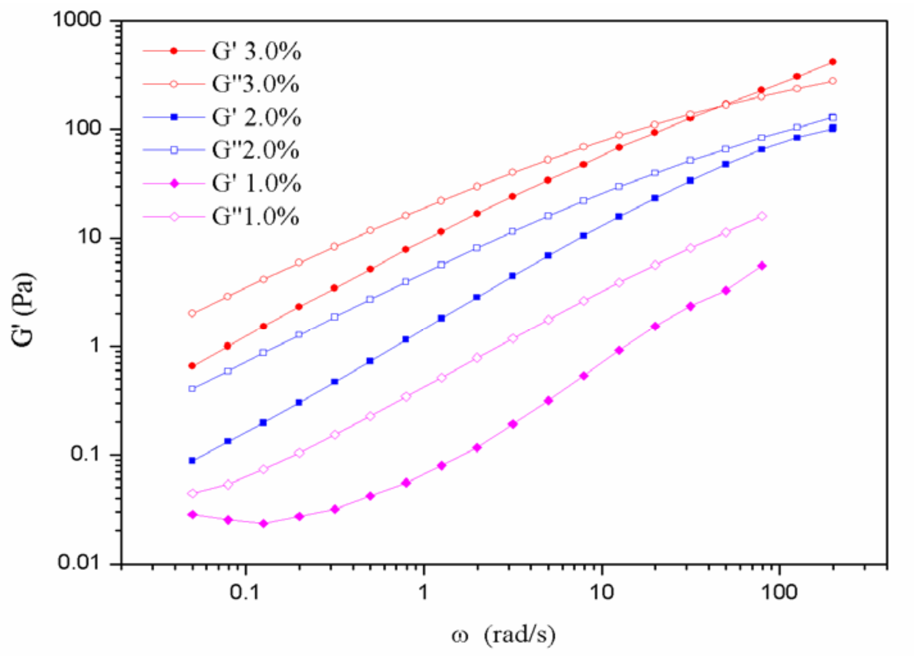

Supplementary Figure S4. Oscillatory frequency sweep measurements showing the storage and loss moduli G′ and G″ as a function of frequency ω for for CS solution with different *c*(CS).

For CS solutions with different *c*(CS), the storage (G′) and loss (G″) moduli both increased with the rise of ω. As mentioned above, the entanglement relaxation time can be determined by the reciprocal of ω at which G′ and G″ curves intersect. Therefore, the entanglement relaxation time was 0.018s of CS solution with *c*(CS)=3.0 wt.%. For CS solution with *c*(CS)=2.0 wt.%, the ω at which G′ and G″ curves intersect was higher than 200 rad/s, and the entanglement relaxation time was shorter than 5×10^{-3}s. For CS solution with *c*(CS)=1.0 wt.%, it could be observed that the intersection of G′ and G″ curves would occur at a frequency higher than that of solution with c(CS)=2.0 wt.%, which indicated even shorter entanglement relaxation time. In conclusion, CS solution with lower *c*(CS) had shorter entanglement relaxation time.

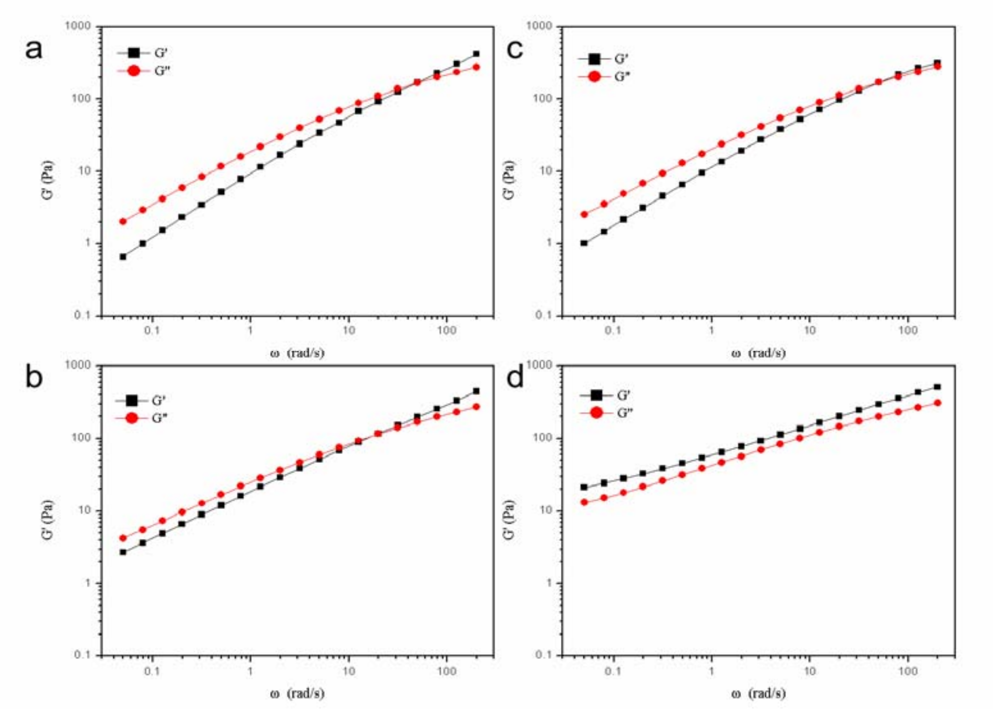

Supplementary Figure S5. Oscillatory frequency sweep measurements showing the storage and loss moduli G′ and G″ as a function of frequency ω for for CS solution at different pH, (a) pH=2.7, unreacted CS solution; (b) pH=3.5; (c) pH=5.5; (d) pH=6.5, CS solution near gelation pH.

For frequency dynamic frequency sweep data presented in Supplementary Figure S10 and Supplementary Figure S11, small-amplitude oscillatory experiments were performed on a AR-G2 rheometer (TA Co., USA) using cone-and-plate geometry. Experiments were run at 20.0 °C. A fixed strain of 2% was used to ensure that the measurements were carried out within the linear viscoelastic range of the samples investigated.

It can be concluded from Supplementary Figure S10, for unreacted solution (Figure S10a), loss moduli (G″) exceeded storage moduli (G′) at lower frequency. The storage (G′) and loss (G″) moduli of CS solution both increased with the rise of ω, and the two curves intersected with each other at ω=55 rad/s. In gel-sol consecutive units, being closer to the source of OH^-, the gap had higher pH. With the increase of pH, the intersection of G′(ω) and G″(ω) appeared at lower frequency (*Supplementary Figure 10b and Supplementary Figure 10c*). Finally, when the system reached gelation point (*Supplementary Figure 10d*), The storage moduli G′(ω) exceeded loss moduli G″(ω). The storage (G′) and loss (G″) moduli of CS solution samples were characterized in the linear viscoelastic region (2%). For the polymer solutions with entanglements, the entanglement relaxation time can reflect by the reciprocal of ω at which G′ and G″ curves intersect

[Yamamoto, T., Ishimura, Y., Takigawa, T. & Masuda, T. Linear viscoelastic properties of concentrated solutions of multi-arm star polystyrenes. *Journal of the Society of Rheology Japan* **31**, 143-148, (2003)]. The oscillatory frequency sweep data indicated that the entanglement relaxation time of unreacted solution is shorter than that of solution near gelation.

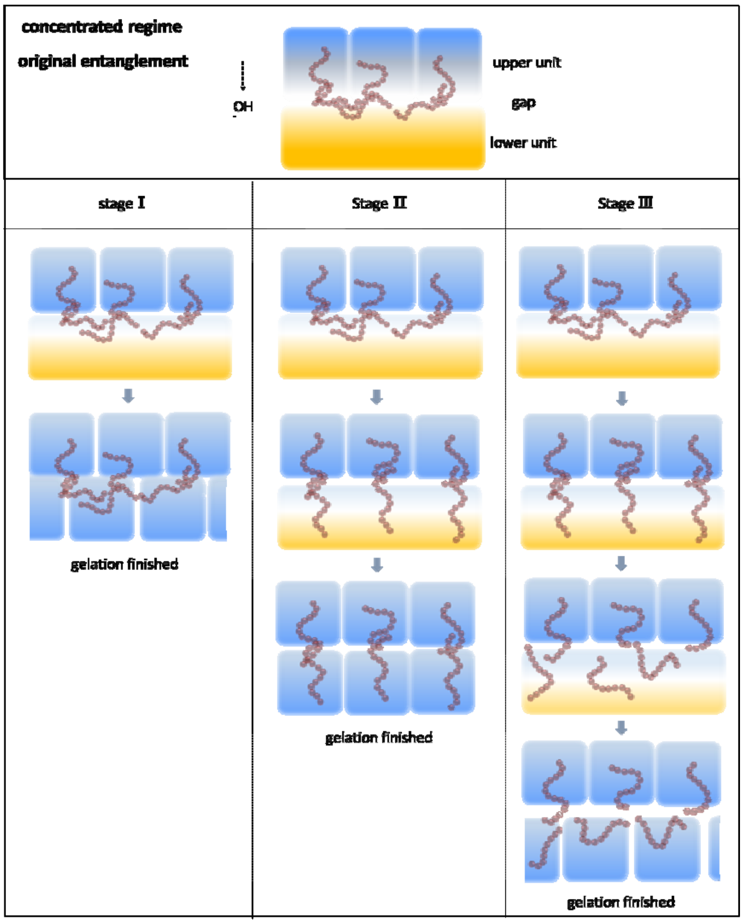

Supplementary Figure S6. Schematic illustration of the three stages in gelation process

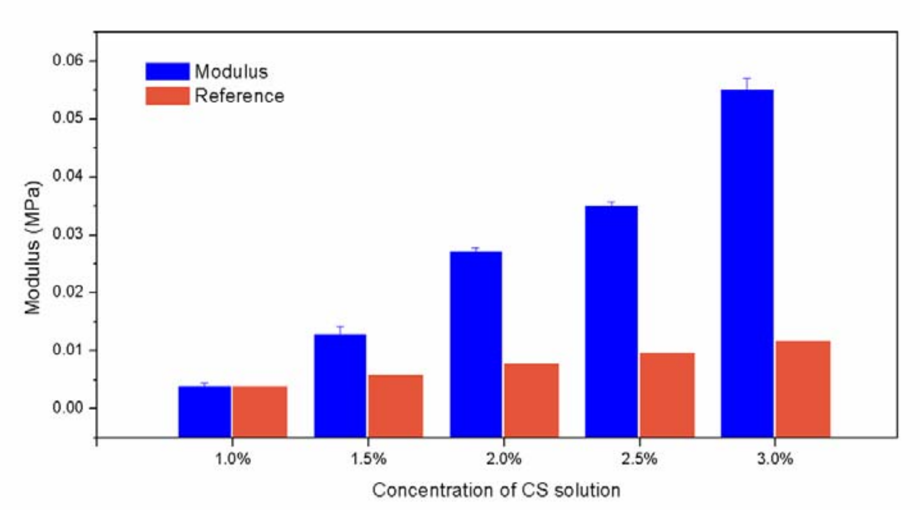

Supplementary Figure S7. Modulus of CS plate shaped hydrogels with different *c*(CS). reference represent the contribution to modulus by the increase of *c*(CS). Error bars indicate standard errors for n=3.

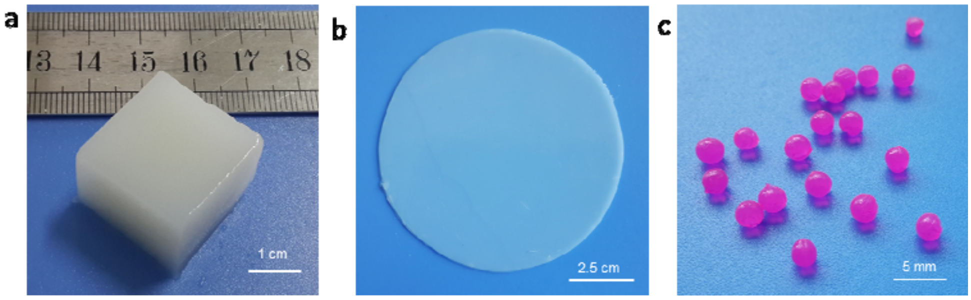

Supplementary Figure S8. Hydrogel with different shapes. (a) Cubic; (b) Membrane; (c) Beads.

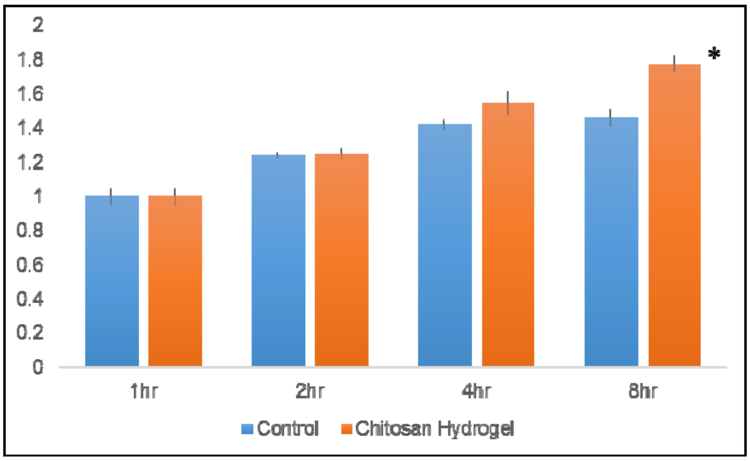

Supplementary Figure S9. The attachment of MC3T3 cells on 96-well plate (control) and chitosan hydrogel for 1 h, 2 h, 4 h and 8 h. (*p<0.05)

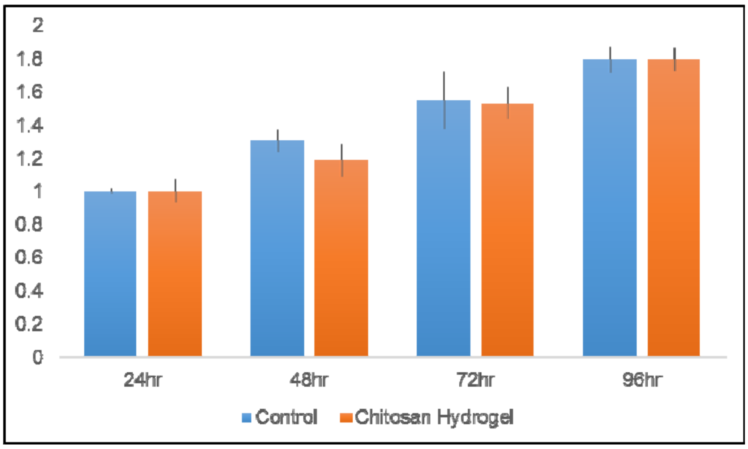

Supplementary Figure S10. Proliferation of MC3T3 cells cultured on 96-well plate (control) and chitosan hydrogel for 24 h, 48 h, 72 h and 96 h. ($p>0.05$)

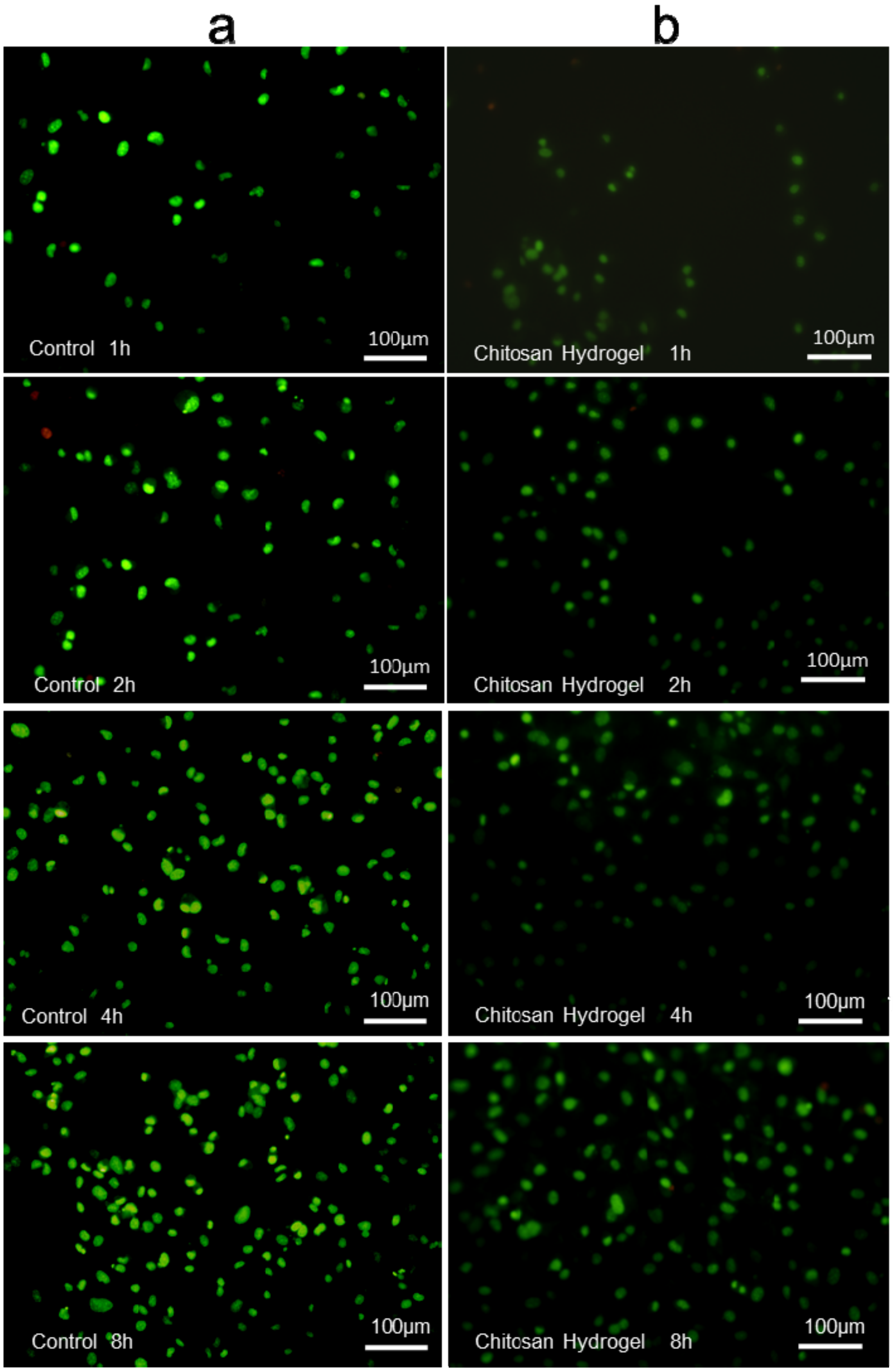

Supplementary Figure S11. Live-Dead Cell Staining of MC3T3 cells cultured for 1 h, 2 h, 4 h and 8 h. (A) Live cells on 96-well plate without chitosan hydrogel. (B) Live cells on chitosan hydrogel.

Cell culture

Chitosan hydrogel were cut into 96-well plate size and left in 75% alcohol overnight, then soaked in α-MEM medium for 24 h. MC3T3 cells were seeded onto a 96-well plate at a density of 2×10^4 cells/well with or without chitosan hydrogel at the bottom. Cells were cultured in complete

α-MEM medium (α-MEM containing 10% heat inactivated FBS and 1% penicillin-streptomycin solution) in a humidified incubator with 5% CO_2 at 37 ^{o}C.

Cell attachment and proliferation

The attachment of MC3T3 cells were assessed at 1 h, 2 h, 4 h and 8 h after cell seeding using CCK-8 kit and Live-Dead Cell Staining kit according to the manufacturer's instructions. The live cells were stained green. The proliferation of MC3T3 cells were also assessed using CCK-8 assay at 24 h, 48 h, 72 h and 9 6h after cell seeding.

Chitosan hydrogel enhance MC3T3 cell attachment

As shown in *Supplementary Figure S9*, the attachment of MC3T3 cells onto chitosan hydrogel was evaluated by CCK8 assay. No difference of cell attachment was observed after 2 hours of cell seeding. However, there was a trend of enhanced cell attachment at four hours. This trend was further enhanced after 8 hours of cell seeding ($P<0.05$), indicating chitosan hydrogel could enhance MC3T3 cell attachment. The enhanced attachment property was further confirmed by Live/Dead cell assay. As shown in *Supplementary Figure S10*, there were increasing numbers of MC3T3 cells on both chitosan hydrogel and culture plate. However, it is noteworthy that the number of MC3T3 cells increased quicker in the chitosan hydrogel group than the cell culture plate, especially between 2 hours and 4 hours. Collectively, these results suggest chitosan hydrogel is cell-friendly and can enhance MC3T3 cell attachment in vitro.

Chitosan hydrogel does not affect MC3T3 cell proliferation

The proliferation of MC3T3 cells on chitosan hydrogel was further investigated by using cell proliferation assay. As shown in Supplementary Figure S11, the proliferative rate of MC3T3 cells was similar on both chitosan hydrogel and cell culture plate at day 1, day 2, day 3 and day 4 respectively. Together, this data suggests chitosan hydrogel does not affect MC3T3 cell proliferation.

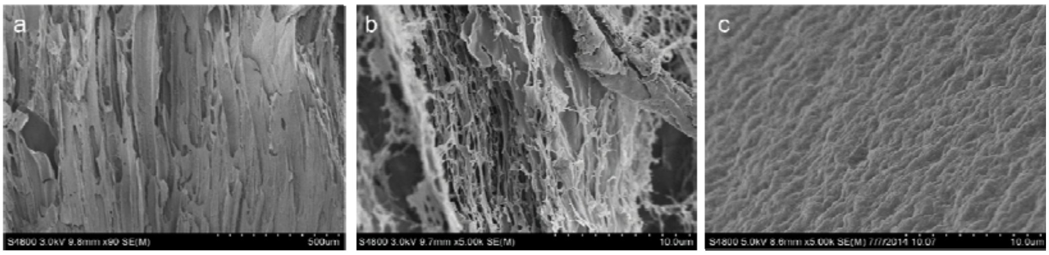

Supplementary Figure S12. SEM images of longitudinal section (a-b), and surface structure (c) of CS hydrogel, *c*(CS)=3.0%.
